# Clinical consequences of switching from olanzapine to risperidone and vice versa in outpatients with schizophrenia: 36-month results from the worldwide schizophrenia outpatients health outcomes (W-SOHO) study

**DOI:** 10.1186/1471-244X-12-218

**Published:** 2012-12-04

**Authors:** Jihyung Hong, Diego Novick, Roberto Brugnoli, Jamie Karagianis, Martin Dossenbach, Josep Maria Haro

**Affiliations:** 1 Personal Social Services Research Unit, London School of Economics and Political Science, Houghton Street, London, WC2A 2AE, UK; 2 European Health Outcomes Research, Eli Lilly and Company, Windlesham, Surrey, UK; 3 Fondazione Italiana per lo studio della Schizophrenia, Rome, Italy; 4 Eli Lilly Canada Inc, Toronto, Ontario, Canada; 5 Eli Lilly Ges.m.b.H, Wien, Austria; 6 Parc Sanitari Sant Joan de Deu, CIBERSAM, Sant Boi de Llobregat, Barcelona, Spain

**Keywords:** Antipsychotic, Schizophrenia, Switching, Olanzapine, Risperidone

## Abstract

**Background:**

With many atypical antipsychotics now available in the market, it has become a common clinical practice to switch between atypical agents as a means of achieving the best clinical outcomes. This study aimed to examine the impact of switching from olanzapine to risperidone and *vice versa* on clinical status and tolerability outcomes in outpatients with schizophrenia in a naturalistic setting.

**Methods:**

W-SOHO was a 3-year observational study that involved over 17,000 outpatients with schizophrenia from 37 countries worldwide. The present *post hoc* study focused on the subgroup of patients who started taking olanzapine at baseline and subsequently made the first switch to risperidone (n=162) and *vice versa* (n=136). Clinical status was assessed at the visit when the first switch was made (i.e. before switching) and after switching. Logistic regression models examined the impact of medication switch on tolerability outcomes, and linear regression models assessed the association between medication switch and change in the Clinical Global Impression-Schizophrenia (CGI-SCH) overall score or change in weight. In addition, Kaplan-Meier survival curves and Cox-proportional hazards models were used to analyze the time to medication switch as well as time to relapse (symptom worsening as assessed by the CGI-SCH scale or hospitalization).

**Results:**

48% and 39% of patients switching to olanzapine and risperidone, respectively, remained on the medication without further switches (p=0.019). Patients switching to olanzapine were significantly less likely to experience relapse (hazard ratio: 3.43, 95% CI: 1.43, 8.26), extrapyramidal symptoms (odds ratio [OR]: 4.02, 95% CI: 1.49, 10.89) and amenorrhea/galactorrhea (OR: 8.99, 95% CI: 2.30, 35.13). No significant difference in weight change was, however, found between the two groups. While the CGI-SCH overall score improved in both groups after switching, there was a significantly greater change in those who switched to olanzapine (difference of 0.29 points, p=0.013).

**Conclusion:**

Our study showed that patients who switched from risperidone to olanzapine were likely to experience a more favorable treatment course than those who switched from olanzapine to risperidone. Given the nature of observational study design and small sample size, additional studies are warranted.

## Background

With many atypical antipsychotics now available in the market, it has become a common clinical practice to switch between atypical agents as a means of achieving the best clinical outcomes [1]. It is, therefore, crucial for clinicians and decision makers to understand the possible benefits and risks associated with switching between atypical agents. Because of the potential implications on prevailing standards of clinical practice, the consequences of switching between olanzapine and risperidone are of particular interest as they are the two most commonly prescribed antipsychotics [2,3].

Some prior research has shown the clinical utility of switching from olanzapine to risperidone or *vice versa*. Ganguli et al. ([2008]) conducted an open-label trial to evaluate the efficacy and safety of risperidone in patients with schizophrenia who had been non-responsive or intolerant to olanzapine. The results of the study showed that switching from olanzapine to risperidone, regardless of whether the switching was abrupt or gradual, was associated with significant symptom improvement, especially in positive and anxiety symptoms, and was generally well tolerated. Clinical benefits of switching to risperidone after insufficient response to olanzapine have also been reported by Takahashi et al. ([2006]b) who focused on patients with first-episode schizophrenia. Conversely, in another open-label trial involving the same type of patients, these authors found that non-responders to risperidone treatment benefited from a switch to olanzapine [4]. The benefit of switching from risperidone to olanzapine was also reported in other open-label trials involving a broader spectrum of patients with schizophrenia [5,6]. While the experimental data from these open-label trials confirmed the benefits of switching between risperidone and olanzapine, patient behavior and outcomes are likely to differ in actual clinical settings. Moreover, except for one costing study [1], there have been no direct comparisons between the switch from olanzapine to risperidone and the switch from risperidone to olanzapine. Using a claims database, Zhao et al. ([2004]) reported that patients who switched from risperidone to olanzapine had a decrease in total medical costs despite an increase in medication-related costs, whereas those switching from olanzapine to risperidone had no significant change in total health care costs despite decreased medication-related costs. Most patients with schizophrenia who change medication do so because they have severe disease that is not fully responsive to the existing medication, so switching to another medication is likely to show improvements. Thus, the relative benefits of two medications can only be fully understood by comparing the changes achieved when switching from one medication to the other and *vice versa*.

To better understand the clinical implications of switching between risperidone and olanzapine in the ‘real world’ setting, the present study conducted a *post hoc* analysis using data accrued from the large naturalistic 3-year W-SOHO (Worldwide-Schizophrenia Outpatient Health Outcomes) study. The specific objectives of the present analysis were two-fold: (1) to compare clinical outcomes before and after switching from oral risperidone to oral olanzapine as well as switching from oral olanzapine to oral risperidone, respectively, and (2) to compare the post-switch clinical outcomes between the two groups.

## Methods

### Study design and patient sample

The Schizophrenia Outpatient Health Outcomes (SOHO) study was a 3-year, international, prospective, observational study primarily designed to assess the comparative costs and outcomes associated with antipsychotic use in outpatients initiating or changing antipsychotic medication for schizophrenia (with an emphasis on olanzapine compared with other antipsychotics). SOHO was conducted in 10 Western European countries (EU-SOHO) [7,8], and in 27 countries across 4 continents as the Intercontinental SOHO (IC-SOHO) [9]. Data from all 37 participating countries were pooled to produce the Worldwide-SOHO (W-SOHO) dataset. A total of 17,384 patients were included in W-SOHO, and the details of the study are available elsewhere [10]. The study was carried out in accordance with the ethical standards of responsible local committees and regulations of the participating countries [7]. It was approved in all countries at the site, regional or national level, depending on the countries’ regulations and participating sites in each country. Patient consent followed country regulations. All patients gave at least oral informed consent and written informed consent was obtained in Denmark, Italy, Portugal, Spain, Ireland, and the UK.

Participating psychiatrists offered enrolment to adult patients (at least 18 years of age) initiating or changing antipsychotic medication for the treatment of schizophrenia, who presented within the normal course of care in the outpatient setting or in the hospital when admission was planned for the initiation or change of antipsychotic medication and discharge was planned within 2 weeks, and who were not participating in another interventional study. The diagnosis of schizophrenia was made by the participating psychiatrists using standard diagnostic criteria [Diagnostic and Statistical Manual of Mental Disorders 4^th^ ed [11] or International Classification of Diseases 10^th^ ed [12]]. Patient enrolment began in September 2000 for EU-SOHO and in November 2000 for IC-SOHO; the last patient was enrolled in December 2001. The enrolment period was intentionally long to avoid interfering with standard medical practice and no minimum number of patients was required per participating psychiatrist.

As the initial objective of SOHO was to compare the outcomes of patients starting olanzapine with other antipsychotics, the study was designed to provide two patient cohorts of approximately equal size: (1) patients starting olanzapine, and (2) patients starting any other antipsychotic. This deliberate over-sampling of olanzapine patients was done to facilitate comparisons between the two groups, in accordance with the primary objective. Importantly, the antipsychotic treatment prescribed to each patient was wholly based on the opinion of the treating psychiatrist; patients were asked to participate in the study after they had received their medication prescription. In addition, patients were not required to continue taking the medication initiated at baseline. Changes in medication, dosing and concomitant medication were possible at any time as determined by the treating psychiatrist.

Data collection for the study occurred at the baseline visit and at follow-up visits (i.e. 3, 6, 12, 18, 24, 30 and 36 months post-baseline) within the normal course of care. Socio-demographic data were recorded at the baseline assessment. Clinical severity was assessed at each visit using a scale based on the Clinical Global Impressions Severity Scale – Schizophrenia version (CGI-SCH) [13], which evaluates symptom severity across positive, negative, depressive and cognitive sub-domains as well as overall symptoms from 1 (normal, not ill) to 7 (among the most severely ill). Other information collected at follow-up visits included clinical status (e.g., weights (kg), alcohol/substance abuse/dependency, suicide attempts, occurrence of violent or aggressive behavior), functional status (e.g., relationships, housing conditions, work and social contacts), antipsychotic medication (drug name, formulation, dosage and reasons for medication change if applicable), concomitant medication (anticholinergics, antidepressant, anxiolytics/hypnotics and mood stabilizers), adverse events, quality of life, and health service use.

### Statistical analysis

Patients with no more than one missing visit (excluding the last visit) were eligible for inclusion in the present analysis (n=11,078, 64% of the baseline sample). For patients with one missing visit, values from the previous visit were carried forward to impute the values of the missing visit. Of the 11,078 study completers, the present study focused on the subgroup of patients who started with olanzapine monotherapy and switched to risperidone monotherapy (n=162) (termed as the ‘OLZ-RIS’ group hereafter) or started with risperidone monotherapy and switched to olanzapine monotherapy (n=136) (termed as the ‘RIS-OLZ’ group hereafter) as the first medication switch during follow-up.

Kaplan-Meier survival curves of the time to (*second*) medication switch were plotted for each group (OLZ-RIS and RIS-OLZ). The overall difference between survival curves was compared using the log-rank test. Medication switch was defined as: (1) stopping the treatment with or without replacing it with another antipsychotic; or (2) adding a new antipsychotic to the treatment. Time to switch was also examined using a Cox proportional hazards model, adjusting for patient characteristics before switching.

The analysis focused on the following key outcomes: change in CGI-SCH overall score; extrapyramidal symptoms (EPS); loss of libido; impotence/sexual dysfunction; amenorrhea/galactorrhea; weight (kg) change; and time to relapse. Outcomes before and after switching were compared within each group using McNemar tests (for categorical variables) and paired t-tests (for numerical variables), except for time to relapse. Outcomes before switching were assessed at the visit when the first switch was made. Outcomes after switching were measured at the visit when a further switching occurred or otherwise at 36-months. Multivariate regression models (logistic or linear regressions) were also used to examine the impact of switching on these outcomes, adjusting for patient characteristics.

Time to relapse was examined only among patients who attained a CGI-SCH overall score of ≤3 (i.e. mildly ill or less) after the first switch from olanzapine to risperidone (or *vice versa*) but before a further switch was made or before 36 months. Starting from this new “baseline”, relapse was defined as an increase of at least 2 points on the GGI-SCH overall severity score from the minimum score achieved by the patient during the follow-up assessment (till a further switch occurred; otherwise till 36-months), resulting in a rating of moderately ill or worse (score ≥4), or having had a hospitalization [14]. Of the 298 patients included, only 186 patients met this criterion (n=98 in the OLZ-RIS group and n=88 in the RIS-OLZ group). Time to relapse for the OLZ-RIS and RIS-OLZ groups was estimated using Kaplan-Meier survival curves, and also analyzed using Cox-proportional hazards model controlling for patient characteristics.

In all multivariate analyses (Cox, logistic, and linear regressions), patient group (i.e. RIS-OLZ group or OLZ-RIS group) served as the main explanatory variable while adjusting for the influence of the following key covariates: time of switch; age; gender; region; and CGI-SCH overall score before switching. Corresponding tolerability outcomes before switching were also included in the analysis of post-switch tolerability outcomes. In addition, other variables before switching were included in models, subject to their significance at p<0.05 through a backward stepwise reduction technique: time since first service contact for schizophrenia; current alcohol abuse/dependency; current substance abuse/dependency; hostility; having a relationship; having paid employment; living independently; being socially active; and taking concomitant medications (anticholinergics, antidepressant, anxiolytics/hypnotics, and mood stabilizers).

All analyses were also repeated in a sensitivity analysis, which defined the *(second)* medication switch as stopping the treatment with or without replacing it with another antipsychotic. That is, in this sensitivity analysis, adding a new antipsychotic to the treatment was not considered as a switching.

## Results

Of the 11,078 study completers, 6,412 patients started with either olanzapine monotherapy (n=4,736) or risperidone monotherapy (n=1,676) at baseline. Of these, a total of 2,493 patients (38.9%) switched their baseline medications over the 3-year follow-up: 1726 (36.4%) among the patients initiated on olanzapine monotherapy and 767 (45.8%) among the patients initiated on risperidone monotherapy. The present study, however, only included a subsample of patients who switched to either olanzapine monotherapy (n=136) or risperidone monotherapy (n=162) as their first medication switch during follow-up. The most common reason for discontinuation was lack of efficacy in both groups (Table 1). The mean dose for olanzapine was 10.1 (SD: 5.3) before switching and that for risperidone was 4.7 (SD: 3.0).

**Table 1:** Medication dose and reasons for discontinuation before and after switching

	**Before switching**		**After switching**	
	**OLZ-RIS (n=162)**	**RIS-OLZ (n=136)**	**OLZ-RIS (n=162)**	**RIS-OLZ (n=136)**
**Dose (Mean ± SD)**	10.1±5.3	4.7±3.0	4.6±3.2	10.9±5.9
**Reason for discontinuation (%)**^**a**^				
Lack of efficacy	41.3	59.1	36.0	34.0
Intolerability	29.7	31.8	15.7	20.0
Lack of compliance	19.4	23.5	19.1	16.0
Patient request	34.8	28.8	19.1	26.0

^a^More than one reason allowed.

OLZ-RIS: Patients who switched from olanzapine to risperidone.

RIS-OLZ: Patients who switched from risperidone to olanzapine.

The demographics and clinical characteristics of the patients before switching are reported for both groups in Table 2. The patient characteristics were similar between the two groups, except that patients in the OLZ-RIS group were significantly more likely to be alcohol dependent/abusive before switching, compared with those in the RIS-OLZ group.

**Table 2:** Patient characteristics before switching from risperidone to olanzapine and from olanzapine to risperidone

	**OLZ-RIS (n=162)**	**RIS-OLZ (n=136)**
Age (years) ^a^	37.2 ± 13.3	36.5 ± 12.5
Male (%)^b^	51.2	51.9
Age at first service contact for schizophrenia (years)^a^	27.6 ± 10.3	27.9 ± 10.5
Duration of illness (years)^a^	9.7 ± 10.1	8.8 ± 8.9
CGI-SCH overall^a^	3.5 ± 1.2	3.6 ± 1.2
CGI-SCH positive^a^	3.0 ± 1.4	3.0 ± 1.4
CGI-SCH negative^a^	3.1 ± 1.3	3.2 ± 1.3
CGI-SCH cognitive^a^	2.8 ± 1.3	3.1 ± 1.3
CGI-SCH depressive^a^	2.7 ± 1.3	3.0 ± 1.4
Current alcohol abuse (%)^b,^*	5.0	1.5
Current substance abuse (%)^b^	2.5	3.7
Being hostile (%)^b^	9.9	11.0
Having a spouse/partner (%)^b^	30.4	24.2
Living independently (%)^b^	55.9	49.6
Being socially active (%)^b^	80.1	77.8
Being employed (%)^b^	29.8	25.2

a Data presented as mean (SD), t-test employed.

b Data presented as %, chi-test employed.

* Significant at p<0.05.

OLZ-RIS: Patients who switched from olanzapine to risperidone.

RIS-OLZ: Patients who switched from risperidone to olanzapine.

CGI-SCH: Clinical Global Impression-Schizophrenia.

**Figure 1:**
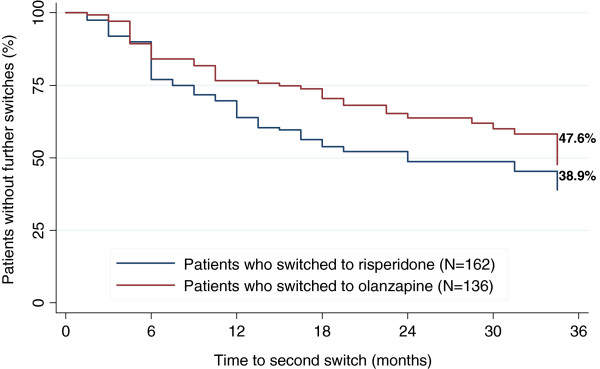
**Kaplan-Meier estimates of time to (*second*) medication switch.** Figure 1 shows the Kaplan-Meier curves for the rate of (*second*) medication switch during follow-up for the OLZ-RIS and RIS-OLZ groups.

Figure 1 shows the Kaplan-Meier curves for the rate of (*second*) medication switch during follow-up for the OLZ-RIS and RIS-OLZ groups. Patients who switched to olanzapine were more likely to remain on this medication longer (47.6% and 38.9% of patients switching to olanzapine and risperidone, respectively, made no further switches, p=0.019). The Cox-regression, which adjusted for differences in patient characteristics before switching, found an increased risk of medication switch in patients who switched to risperidone, compared with those who switched to olanzapine, although this was not statistically significant at the 0.05 level (HR= 1.44; 95% CI=0.97, 2.12; p=0.070).

Patients who started treatment with either olanzapine or risperidone experienced symptom improvement on their initial medication (as assessed by the CGI-SCH overall score), and a further improvement after the medication switch (Table 2). Patients in the RIS-OLZ group achieved a mean reduction in CGI-SCH overall score from baseline of 0.71 points (SD: 1.20) and 1.46 points (SD: 1.29) before and after the medication switch, respectively. Likewise, patients in the OLZ-RIS group achieved a mean reduction from baseline of 0.75 points (SD: 1.05) and 1.15 points (SD: 1.12) before and after the medication switch, respectively.

**Figure 2:**
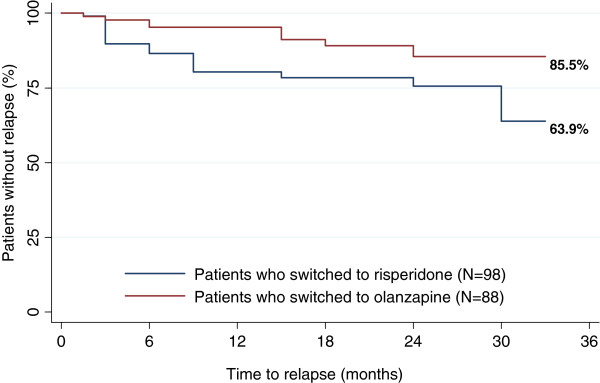
**Kaplan-Meier estimates of time to relapse.** Figure 2 shows the Kaplan-Meier curves for the rate of relapse during follow-up for the OLZ-RIS and RIS-OLZ groups. This analysis was confined to those patients who attained a CGI-SCH overall score of ≤3 (i.e. mildly ill or less) after the first switch from olanzapine to risperidone (or *vice versa*) but before a further switch was made or before 36 months (n=98 in the OLZ-RIS group and n=88 in the RIS-OLZ group).

While both groups showed clinical improvement after switching, tolerability outcomes differed between the two groups (Table 3). Patients who switched to olanzapine experienced significant improvements after switching in all tolerability outcomes except weight change, which did not differ significantly between before and after switching. Patients who switched to risperidone did not make improvements in any of the tolerability outcomes after switching and experienced a worsening in amenorrhea/galactorrhea. In addition, the Kaplan-Meier curves showed that 85.5% of patients in the RIS-OLZ group did not experience relapse during follow-up, while only 63.9% of patients in the OLZ-RIS group did not relapse during follow-up (p=0.015) among patients who attained a CGI-SCH overall score ≤3 (i.e. mildly ill or less) (Figure 2). Consistent with this, the Cox-regression model showed that patients who switched to risperidone were more likely to experience relapse during follow-up (HR=3.43; 95% CI= 1.43, 8.26; p=0.006), compared with those who switched to olanzapine.

**Table 3:** Change in clinical status before and after switching

	**Before switching**		**After switching**	
	**OLZ-RIS (n=162)**	**RIS-OLZ (n=136)**	**OLZ-RIS (n=162)**	**RIS-OLZ (n=136)**
**Change in CGI-overall score from baseline**^**a**^**(Mean ± SD)**	−0.75±1.05	−0.71±1.20	−1.15±1.12	−1.46±1.29
**EPS**^**b**^**(%)**	15.2	28.4	15.2	9.0
**Loss of libido**^**b**^**(%)**	28.8	37.8	31.3	26.7
**Impotence/sexual dysfunction**^**b**^**(%)**	18.9	29.3	22.0	19.6
**Amenorrhoea/galactorrhea**^**a**^**(%)**	12.0	12.6	17.7	5.9
**Change in weight (kg) from baseline**^**c**^**(Mean ± SD)**	3.14±6.05	1.43±4.21	4.11±8.39	1.95±8.90

^a^The difference between before and after switching was significant in both groups (p<0.05).

^b^The difference between before and after switching was significant only in patients who switched to olanzapine (i.e., the RIS-OLZ group).

^c^The difference between before and after switching was not significant in either group.

OLZ-RIS: Patients who switched from olanzapine to risperidone.

RIS-OLZ: Patients who switched from risperidone to olanzapine.

EPS: Extrapyramidal Symptom.

Table 4 summarizes the results of the regression analyses. Linear regression showed improvements in the CGI-SCH overall score after switching in both groups, and that those who switched to olanzapine had a further 0.29 point reduction in this score (p=0.013). Logistic regression analyses also showed more favorable outcomes in the RIS-OLZ group after switching, compared with the OLZ-RIS group: patients who switched to risperidone were more likely to have EPS (OR=4.02; 95% CI=1.49, 10.89; p=0.006) and amenorrhea/galactorrhea (OR=8.99; 95% CI=2.30, 35.13; p=0.002). There were no statistically significant differences between the two groups after switching for the other tolerability outcomes (loss of libido, impotence/sexual dysfunction and weight change).

**Table 4:** The impact on clinical outcomes of switching to risperidone, compared with switching to olanzapine

	**Estimate**	**95% confidence interval**	**P-values**
**Cox regression**	Hazard ratio^a^		
Medication switch	1.44	0.97, 2.12	0.070
Relapse	3.43	1.43, 8.26	0.006
**Linear regression**	Coefficient^b^		
Change in CGI-SCH overall score before and after switching	0.29	0.06, 0.52	0.013
Change in weight (kg) before and after switching	0.22	−1.37, 1.81	0.785
**Logistic regression**	Odds ratio^c^		
EPS	4.02	1.49, 10.89	0.006
Loss of Libido	1.77	0.93, 3.37	0.083
Impotence/sexual dysfunction	1.93	0.95, 3.94	0.070
Amenorrhea/galactorrhea	8.99	2.30, 35.13	0.002

^a^A hazard ratio of more than 1 indicates an increased risk of medication switch or relapse, compared with patients switched to olanzapine.

^b^Linear regression modeled a change in CGI-SCH overall score (or weight) before and after switching. Coefficients indicate an estimated difference in the change, compared with patients switched to olanzapine.

^c^An odds ratio of more than 1 indicates an increased likelihood of having tolerability outcomes compared with patients switched to olanzapine.

The results of the sensitivity analysis (where adding a new antipsychotic to the treatment was not considered as a medication switch) were largely consistent with these findings (results available upon request).

## Discussion

To our knowledge, this is the first study that has assessed and compared clinical outcomes (effectiveness/tolerability) of switching from olanzapine to risperidone and *vice versa* in the usual course of schizophrenia care. Our results showed that patients who were treated with risperidone and subsequently switched to olanzapine for clinical reasons experienced a more favorable treatment course than patients who were treated with olanzapine and subsequently switched to risperidone. Patients who switched to olanzapine experienced significant improvements in symptom control (as assessed by the CGI-SCH overall score) and in all tolerability outcomes (EPS, loss of libido, impotence/sexual dysfunction, and amenorrhea/galactorrhea) except for weight gain. On the other hand, patients who initiated with olanzapine and switched to risperidone did not experience improvements in any of the tolerability outcomes, although they experienced significant improvements in symptom control. The differential effect between the two groups remained even when the patient characteristics before switching were taken into account in multivariate analyses. Patients who switched to olanzapine were more likely to improve in symptom control and less likely to experience relapse, EPS and amenorrhea/galactorrhea, compared with those who switched to risperidone.

Consistent with previous research [5,15], patients experienced symptom improvement after medication switch in both the RIS-OLZ and OLZ-RIS groups. However, symptom improvement was greater in those who switched from risperidone to olanzapine. This finding was partially in agreement with recent results from the Clinical Antipsychotic Trials for Intervention Effectiveness study (CATIE) [16]. In phase 2T of the CATIE study (conducted in patients who primarily experienced intolerability to their previous antipsychotic), the olanzapine group achieved greater improvement than the risperidone group in positive symptom control as assessed by the Positive and Negative Syndrome Scale (PANSS). However, there were no differences between groups in negative and overall symptoms. Data from other head-to-head trials are available [17], but they do not suggest that one drug is clearly more effective than the other in symptom control. However, consistent with our findings, olanzapine appears to be superior to risperidone in relapse prevention, EPS and reproductive adverse events [17].

Relapse prevention is a primary goal in the long-term treatment of schizophrenia. Surprisingly, however, there is only limited information on the comparison between risperidone and olanzapine. Nevertheless, this small body of literature also reports a lower risk of relapse in patients treated with olanzapine compared with those treated with risperidone [14,18,19].

EPS are common adverse events during treatment with antipsychotics, especially for conventional agents. They are the most common reason for non-adherence with antipsychotic medication, leading to treatment failures, relapses, poorer long-term outcomes and preventable economic costs [20-22]. Atypical antipsychotics have a lower risk of EPS than conventional antipsychotics, and this is a considerable asset from a tolerability and safety point of view [23]. However, the risk of EPS varies among atypical antipsychotics and dose-dependent increases in EPS have been observed with risperidone [24]. Several studies have provided the evidence that patients treated with olanzapine may have a lower risk of EPS than patients treated with risperidone [25]. Previous SOHO publications also showed a lower risk of EPS with olanzapine compared with risperidone [26]. Moreover, a UK population-based study reported reduced use of antiparkinsonian drugs (treatment for EPS) in patients who switched from conventional agents to olanzapine, but not in those who switched to risperidone [20]. A reduced use of antiparkinsonian drugs was also observed in patients who switched from risperidone to olanzapine, but not in those switched from olanzapine from risperidone [1]. Lowering the risk of EPS is crucial in the management of schizophrenia, given their clinical and economic implications.

Consistent with previous studies [27,28], our study demonstrated a lower risk of amenorrhea/galactorrhea with olanzapine compared with risperidone. One possible reason for this may be the potent effect of risperidone on prolactin elevation, although plasma prolactin concentrations were not measured in the SOHO study. Prolactin elevation is a well-known adverse event of conventional antipsychotics and of some atypical antipsychotics such as risperidone [29], although treatment with olanzapine may also elevate prolactin level and the elevation may persist during chronic administration [30]. An increase in prolactin levels can cause amenorrhea, galactorrhea and other sexual disturbances [29,31]. A number of studies have shown that the risk of prolactin elevation with risperidone is similar to or greater than that of conventional antipsychotics [29,31,32]. The switching study from olanzapine to risperidone by Takahashi and colleagues also reported a significant increase in plasma prolactin concentrations after the medication switch [33]. However, the findings of a recent study in patients with first episode psychosis indicated that such reproductive adverse events can occur even when prolactin levels are normal [34]. While the mechanisms underlying such reproductive adverse events in antipsychotic-treated patients are not yet clearly understood, it is well known that hyperprolactinemia can cause amenorrhea and galactorrhea.

In addition, weight gain has been commonly reported during treatment with most atypical antipsychotics, including olanzapine and risperidone [29]. However, our study did not find any significant differences in weight change before and after medication switch between the two groups (i.e. RIS-OLZ and OLZ-RIS groups). Notably, however, patients who were on olanzapine at baseline gained an average of 3.14 kg before switching to risperidone. In comparison, those who were initially on risperidone gained an average of 1.43 kg before switching to olanzapine.

The results of this study also confirmed that medication switch is a common practice in the management of schizophrenia [29]. More than one in three outpatients with schizophrenia who initiated treatment with either olanzapine or risperidone switched antipsychotic medication at least once during the 3-year follow-up in the normal course of care. In addition, more than half of the switchers also made subsequent switches (52.4% for patients who switched to olanzapine and 61.1% for patients who switched to risperidone). While medication switch constituted a logical and common treatment strategy for patients who did not respond adequately to the prescribed antipsychotic treatment or could not tolerate treatment-emergent adverse events, it may not always result in improvement, as indicated in our study. Although the results of our study confirmed that switching to olanzapine would be of benefit to patients who failed treatment with risperidone, the converse was not confirmed.

Our results need to be interpreted in the context of the following study limitations. Firstly, the W-SOHO study was originally designed and powered to compare clinical and economic outcomes between olanzapine and other antipsychotics. Comparison of the impact of switching antipsychotics between olanzapine and risperidone was a *post hoc* analysis and, consequently, the relevant subgroup sample size was small. This, in turn, might have affected the statistical results. Secondly, our study sample was initially drawn from 11,078 patients, who completed the study and had no more than one missing visits during follow-up (i.e., 64% of the baseline sample). Although the retention rate of 64% was relatively high given the study duration of three years and thereby the same size of study completers was still very large, the clinical prognosis could differ between study completers and those who were not. It is, however, unclear whether the inclusion of study completers only disproportionally influenced the outcomes of the OLZ-RIS and RIS-OLS groups. Thirdly, as the outcome assessments were not performed blind, we cannot exclude the possibility that the treating psychiatrists were likely to evaluate patients more favorably after the switch. Nevertheless, this bias is unlikely to differ between medications. In addition, as the clinical outcomes were assessed by the treating psychiatrists, the assessment could have been subjective and influenced by their prior knowledge and expectations (i.e., observer bias). This could be of particular concern in the absence of blinding if more favourable assessments were made towards their preferred treatment. Notably however, a previous study [35] explicitly investigated the observer bias in SOHO by comparing patient- and investigator-reported outcomes, and found no such bias. Fourthly, although our findings were adjusted for pre-switch clinical and demographic characteristics of patients when comparing the impact of switching on clinical outcomes, there could be unobserved differences between the treatment groups, which may confound our results. Finally, for patients who switched medication between visits, we assumed that clinical status was similar to that at the visit before the switch.

## Conclusion

The results of the present study demonstrate that there is a more favorable treatment course in patients with schizophrenia who switched from risperidone to olanzapine. They were significantly more likely to remain on the medication longer and improve in symptom control, and significantly less likely to experience relapse, EPS and amenorrhea/galactorrhea, compared with those switched from olanzapine to risperidone. This suggests that while olanzapine can be an effective treatment option for patients requiring a switch from risperidone, treatment with risperidone may have limited benefits for those requiring a switch from olanzapine. Nevertheless, given the nature of observational study design and small sample size, additional studies are warranted.

## Competing interests

JH is a consultant for Eli Lilly and Company. DN, JK and MD are employees of Eli Lilly and Company. Josep Maria Haro received honoraria for lecturing and consultancy (including attending advisory boards) from Eli Lilly and Company, AstraZeneca, and Lundbeck. Roberto Brugnoli received honoraria for lecturing and consultancy from the manufacturers of several antipsychotic agents including Eli Lilly and Company.

## Authors’ contributions

JH conducted the statistical analysis and drafted the manuscript. DN, RB, JK, MD and JMH participated in the design of the present study and provided critical review of the manuscript. All authors have read and approved the final manuscript.

## Pre-publication history

The pre-publication history for this paper can be accessed here:

http://www.biomedcentral.com/1471-244X/12/218/prepub
